# The relationship between locus of control and pre-competitive anxiety in highly trained soccer players

**DOI:** 10.3389/fpsyg.2023.1227571

**Published:** 2023-08-03

**Authors:** Imen Ben Amar, Chiraz Gomni, Oussama Gaied Chortane, Aymen Khmiri, Rania Ghouaiel, Julien S. Baker

**Affiliations:** ^1^High Institute of Sport and Physical Education, Manouba University, Tunis, Tunisia; ^2^Research Unit Sports Performance, Health and Society, ISSEP Ksar Saïd, Manouba, Tunisia; ^3^Philab Laboratory of Cultures, Technologies, and Philosophical Approaches, Tunis, Tunisia; ^4^Centre for Health and Exercise Science Research, Hong Kong Baptist University, Kowloon, Hong Kong SAR, China

**Keywords:** precompetitive anxiety, locus of control, internal locus, external locus, elite athletes

## Abstract

**Introduction:**

Previous studies have not considered the potential association between locus of control and precompetitive anxiety in elite soccer players. Accordingly, this cross-sectional study examined The prediction of locus of control on precompetitive anxiety in highly trained cadet soccer players.

**Objective:**

Based on a literature review, our research question was: can the locus of control be considered as an explanatory element of precompetitive anxiety?

**Methods:**

Thirty-five Tunisian highly trained soccer players licensed from two regional soccer clubs aged between 15 and 16 years participated in the resent study. All participants were evaluated using the Competitive State Anxiety Inventory-2 (CSAI-2) and the Internal-External Locus of Control Scale. The relationship between measures of anxiety, self-confidence and the locus of control scores were analyzed using Pearson’s product–moment correlation coefficient. Further, multiple linear stepwise multiple regression models were calculated to determine the most robust predictors of the locus of control.

**Results:**

Based on our findings, the regression analysis explains up to 21.3% of the total variation of our independent variable (locus of control) and explains only 21.3% of the variability of our dependent variable somatic anxiety. Furthermore, locus of control explains 61.9% of the variability in self-confidence.

**Conclusion:**

The locus of control can be used for the detection and selection of young athletic talent to identify individuals with the best psychological aptitude to cope with psychological problems related to sports performance. Preparing highly trained soccer players on how to deal with their anxiety could prevent them from becoming overwhelmed when they feel powerless to change their situation during competition.

## 1. Introduction

Identifying performance factors in soccer is a complex process. It has been pointed out that soccer performance complexity is reflected by difficulty in combining different performance factors: these include technical, tactical, physical and psychological (Boulogne, 1979; Slimani and Nikolaidis, 2017). Psychological factors constitute an inherent part of performance variables (Reilly and Gilbourne, 2003). In this context, they must be subject to a scheduled program which is interdependent of other tactical, technical and physical components (Hoff, 2005). In this regard, sports psychology is a branch of psychology investigating the scientific study of individuals and their behavior in a sports or physical activity context. One of the purposes of sport psychology is to provide an understanding of emotional stabilization. As a result, positive and negative emotions are always an area of interest (Martinent et al., 2015). In the sporting context, certain feelings that have produced a particular experimental interest include feelings of “stress,” “pressure,” “tension,” “fear of competition,” “tenseness,” “concern” and “fright.” In sports psychology these emotions have been defined as varying states of anxiety. These emotions are a major study area in sports psychology, and incorporate unpleasant sensations and consequences on performance, in both favorable or unfavorable ways, and are debated and discussed among psychologists (Burton and Naylor, 1997). Also, improving performance has been a fundamental necessity for all highly trained soccer players who wish to excel in their sports careers. Highly trained soccer players train throughout the year to increase their performance potential. Unfortunately, this high-performance expectation also increases the level of anxiety in highly trained soccer players during performance (Coakley and Pike, 2009).

Emotions in sport are important charactersitcs for performance outcomes and an understanding of emotions can sometimes be the difference between winning or losing. It is more often the case that an athlete experiences underperformance due to emotional and mental aspects rather than due to technical or physical performance characteristics. Furthermore, it is quite normal that comparisons have been made between the theory of the locus of control and that of causal attributions. The locus of control refers to an individual’s perception about the underlying main causes of events in his/her life. Or, more simply, do individuals believe that outcomes in life events are controlled by the individual or by external forces. Numerous studies reveal the existence of a negative relationship between the locus of control and anxiety (Rossier et al., 2002, 2009). In a non-sporting context, it has been demonstrated that a positive correlation exists between externality factors and anxiety and a negative correlation between internal beliefs and anxiety in health (Lee et al., 2014).

The study by Polman et al. (2007) investigated young competitive swimmers. They measured the impact of the causal attributions on sports performance and observed that children who won their race had a preferential internal attribution. In addition, the researchers found in the same children, lower levels of cognitive anxiety (not found with somatic anxiety). Another study investigated the relationship between the locus of control and competitive anxiety. The study involved 150 high-level tennis players. The findings of the study demonstrated that the players had a belief in internal control. The decrease in anxiety demonstrated improved sports performance and confirmed the link between belief and the feeling of control for competitive anxiety (Arnaud et al., 2012). Furthermore, the association of physical activity with anxiety is widely documented in the literature (St-Pierre et al., 2019). The aferomentionned studies showed an inverse relationship between anxiety and healthy behaviors, including physical activity. Chirico et al. (2020) evaluated an integrated model linking both Self-determination theory variables and anxiety in relation to physical activity. In addition, according to García-Secades et al. (2017), when the recovery resources start to be much less than the requirements posed by hard conditions, a bad cycle could start for the athlete which might result in a rupture of homeostasis, pressing him/her to a continuous increase in anxiety levels. Full recovery is therefore allowed by a functional synergy of the relational component with the internal component of orientation and focus on the objective. Although the relationship between anxiety and the locus of control have been the subject of numerous studies in sports psychology, the diversity of the results obtained makes it difficult to conclude as to the precise nature of the relationship, particularly in relation to the existence of different forms of anxiety (cognitive anxiety, somatic anxiety, state anxiety, trait anxiety) as well as the diversity of the methods used to measure the traits.

Given the dearth of studies investigating the association between locus of control and precompetitive anxiety in elite athletes, the objective of the present study was to investigate any relationships between precompetitive anxiety and locus of control in highly trained soccer players. We hypothesized that pre-competitive anxiety is predicted by the level of the subject’s locus of control, and that highly trained soccer players with an internal locus of control are less anxious than participants with an external locus of control.

## 2. Materials and methods

### 2.1. Participants

Our study focused on 35 Tunisian highly trained soccer players licensed from two regional soccer clubs. All were cadets and their ages ranged between 15 and 16 years of age (Table 1). Legal guardians and participants provided written informed consent following a thorough explanation of the objectives and scope of the research project, including the procedures, risks, and benefits of the study. The study was conducted according to the latest version of the Declaration of Helsinki, and the protocol was fully approved by the Local Ethics Committee before the commencement of assessments. None of the participating highly trained soccer players had a history of neurological, or orthopedic disorders that may have impaired their applied tests.

**Table 1:** Descriptive statistics of the applied parameters in the study participants.

	**Nombre (*N*)**	**Minimum**	**Maximum**	***M***	**SD**
Locus of control	35	6	21	13.83	5.21
Congnitive anxiety	35	13	34	21.49	7.59
Somatic anxiety	35	8	26	15.26	4.49
Self-confidence	35	11	35	24.63	7.45

### 2.2. Experimental design

Tests were carried out at the same time of day (between 5 p.m. and 7 p.m.) and under similar environmental conditions (temperature: 18–22°C, humidity: 50–60%, Wind speed: ≤2 m/s). All participants were encouraged to provide maximum attention throughout testing. One week before the commencement of the study, two orientation sessions were held to familiarize participants with the general environment and measurement procedures to minimize subsequent learning effects. All participants were tested for the Competitive State Anxiety Inventory-2 (CSAI-2) and the Internal-External Locus of Control Scale.

### 2.3. Anxiety and self-confidence tests

#### 2.3.1. Anxiety and self-confidence tests

Before the intervention started, all participants used the CSAI-2 [7] to evaluate the emotional adaptation to the multidimensional constructs of cognitive anxiety, somatic anxiety, and self-confidence, using a total of 23 items (7 for cognitive anxiety and for somatic anxiety, and 9 for self-confidence). The reliability and validity of the translation of the CSAI-2 using 13 items were reported previously by Boudhiba et al. (2015) [38] with *α* = 0.85. The CSAI-2 was used as it has been validated for the population under investigation. Symptom intensity levels were rated on a 4-point Likert scale ranging from 1 (‘not at all’) to 4 (‘very much so’). A high score on the self-confidence scale implied confidence in one’s ability to deal with the competitive situation in question. A high score on somatic anxiety scale reflected the perception of many psychological reactions to this challenge, and a high score on cognitive anxiety scale indicated difficulties in concentration and negative concerns about performance. The Cronbach alpha coefficients of items relating to all variables are good (range: 0.83 to 0.89) [38], indicating an acceptable level of internal consistency.

### 2.4. The internal-external locus of control scale

The instrument used was Rotter’s (1966) Internal-External (I-E) Locus of Control Scale. The higher the test score, the more external the person’s orienta-tion, with the range of scores being 0 to 23. Comprehensive reviews reporting reliability and validity data on the I-E scale have been completed by Rotter (1966) and Lefcourt (1966). In these reviews the test–retest reliability was acceptable, varying between 0.49 and 0.83 for college freshmen, at time intervals ranging from one to two months. Several investigators have reported on the most recent research on the I-E scale (Hersch and Scheibe, 1967; Joe, 1971; Lefcourt, 1972; Gozali et al., 1973). Hersch, Scheibe and Gozali et al. studies reported test–retest reliabilities generally in the 0.70’s and 0.80’s for college students. Several studies have been undertaken to establish convergent validity (Blackman, 1962; Cardi, 1962; Adams-Webber, 1969), with evidence for the convergent validity of the I-E scale being found in each case. Blackman (1962) used college students and studied the factors affecting the perception of events, as to whether they were chance, or skill determined. Thus, he independently assessed the students’ locus of control through their scores on the perception test. When Blackman compared the students’ scores on the I-E scale with their scores on the perception test he obtained a cor-relation of 0.56. Cardi (1962), also used college students, and measured locus of control independently through a semistructured interview. Judges’ ratings were correlated with I-E scores obtained earlier. A biserial correlation of 0.61 was found for subjects rated high or low from the interview with their I-E scores. Finally, Adams-Webber (1969) independently measured locus of control through the number of external endings for story completions his subjects gave. The subjects were divided into groups, based on the number of external endings given. Those subjects who gave a larger number of external endings tended to be those with external scores on the I-E scale. Adams-Webber found a highly significant difference among the groups (*p* < 0.05).

The discriminant validity of the I-E scale is indicated by low relationships with such variables as intelligence, social desirability, and political liberalness (Rotter, 1966).

### 2.5. Statistical analyses

All statistical analyses were performed using the software statistical package (SPSS Inc., Chicago, IL, version. 16.0). Normal data distribution was tested and confirmed using the Shapiro Wilk test. Parametric statistical tests were applied, and data were presented as means and standard deviations (SD). Test-re-test reliability of our variables was computed using Cronbach’s model of ICCs and SEMs in accordance with the method introduced by Hopkins (Calatayud et al., 2014). The standard error of measurement (SEM) was estimated through the usual formula: SEM = SDd/√2 (Thomas et al., 2022). The relationship between measures of anxiety, self-confidence and the locus of control scores was analyzed using Pearson’s product–moment correlation coefficient. Associations are reported by their correlation coefficient (r-value), level of significance (value of p), and the amount of variance explained (r^2^-value). Values of r ≥ 0.10 indicate small, r ≥ 0.30 medium, and r ≥ 0.50 large size of correlation (Hopkins, et al., 2009). Further, multiple linear stepwise multiple regression models were calculated to determine the most robust predictors of the locus of control. Coefficients of determination (R2 × 100) were used to interpret the meaningfulness of the relationships (Calatayud et al., 2014).

## 3. Results

All participants received treatment conditions as allocated all completed the study measurements with a mean adherence rate of 100%. Table 1 displays test data for all components of the dependant variables measured. Table 2 displays the test–retest reliability analyses for all parameters. ICCs showed good reliability for all anxiety, self-confidence, and locus of control score. Furthermore, a paired t-test showed no significant differences between the scores recorded during the two trials for all measured variables.

**Table 2:** Test–retest reliability of the applied questionnaire and variables.

	Alpha de cronbach	*N*
Locus of control	0.885	23
Congnitive anxiety	0.895	9
Somatic anxiety	0.703	9
Self-confidence	0.897	9

### 3.1. The association between somatic and cognitive anxiety, self-confidence and locus of control

#### 3.1.1. Somatic and cognitive anxiety

The regression of the points were related linearly and the adjustment was of good quality as measured by the coefficient of determination (*R*_2_ = 0.748) which outlines that the correlation is high enough to use the model for predictive applications. On the other hand, the line of adjustment depends on the shape of the cloud passing in the middle of the points. The sequence moves from the lower left to the upper right. This indicates that cognitive anxiety and the locus of control evolved in the same way.

**Figure 1:**
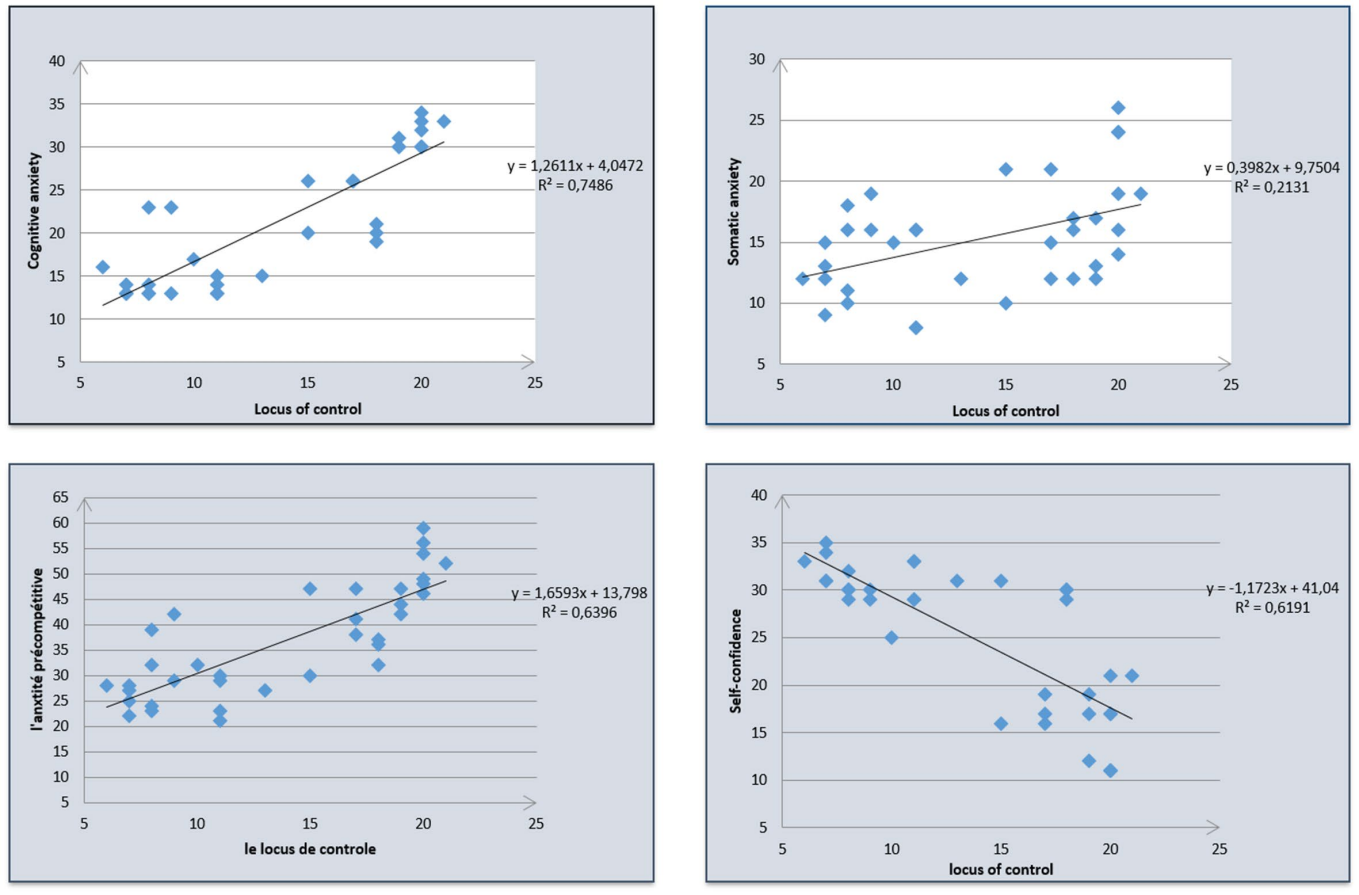
Predictors of locus of control in the sample of elite soccer players.

The regression analysis explains up to 21.3% of the total variation of our independent variable (locus of control) and explains only 21.3% of the variability of our dependent variable somatic anxiety. The F ratio in the ANOVA table examines whether the overall regression model fits the data well. The table shows that the independent variables statistical prediction *F* (1.33) = 8.939 is significant at 0.005 and *p* < 0.05. The points are closer linearly, we then observe that the adjustment is of good quality, measured by the coefficients of determination (*R*_2_ = 0.639) suggesting that the correlation observed was average (Figure 1).

#### 3.1.2. Self-confidence

The coefficient of determination measures part of the variation of Y. We can see from our value of 0.619 that the regression explains up to 61.9% of the total variation of Y. Our independent variable (locus of control) explains 61.9% of the variability of our dependent variable (self-confidence).

The F ratio in the ANOVA table tests whether the overall regression model fits the data well. The table shows that the independent variables statistically predict *F* (1.33) = 53.634 is significant at 0.001 and *p* < 0.05, which indicates that we have less than 0.1% chance of being wrong. The sign of beta (−) therefore the relationship between the two variables is negative. Furthermore, the level of locus of control negatively predicts self-confidence. According to the dispersion of the points around the line, we find that the fit is of good quality measured by the coefficients of determination (*R*_2_ = 0.619) which explains why the correlation is high enough for the model to be used for predictive applications. On the other hand, it is quite possible to extend a line of adjustment which depends on the shape of the cloud by passing through the middle of the points of which this line goes from the upper left to the lower right. This proves that self-confidence and locus of control evolve in opposite directions and therefore the relationship is negative (Figure 1).

## 4. Discussion

The objective of this study was to verify if there was a relationship between precompetitive anxiety and the locus of control and if the nature of this relationship was weak or strong in highly trained cadet soccer players. We verified the prediction of locus of control using the three dimensions of the CSAI-2 questionnaire. Based on the results of linear regression analyses, we found that the level of locus of control positively and significantly explains somatic anxiety in only 21% of the population which means that when somatic anxiety increases, the locus of control also increases. The low value of the correlation coefficient must be considered, and we can partly explain this as being related to the competition environment (being on the track, noises from the spectators, the direct contact with the competitors…) all these factors will cause somatic-anxiety manifestations in the study participants.

Interestingly, anxiety acted as a moderator in the relation between autonomous motivation and physical activity behavior. In detail, the direct effect of autonomous motivation on the implementation of the behavior was stronger in participants with low anxiety than in athletes with high levels. Moreover, the results show that high levels of anxiety boost the intention effect of enacting this behavior. In other words, anxiety may boost physical activity depending on its levels, which moderate the reciprocal effects as outlined. To speculate, our results show that anxiety influences the adoption of physical activity behavior through two different pathways: in the first, participants with low anxiety are keener to keep their physical routine, satisfying their needs as a direct effect of the pleasure to enact the behavior; while in the second, a high level of anxiety seems to “activate” a more volitional–intentional behavior, most likely as a coping strategy. In fact, the literature shows that individuals may adopt physical activity in order to reduce anxiety levels, bringing pleasure and relief [38]. These elements are found more in the competitive context. This may be the reason that explains the lower somatic anxiety scores in the pre-competitive environment. Thus, the locus of control positively and significantly predicts cognitive anxiety 74% (a strong relationship), this explains that an increase in cognitive anxiety occurs simultaneously with an increase in the locus of control.

So, prior to competition, highly trained soccer players who perceive the competition as threatening and have doubts about themselves and their skills as well as the competition environment constitutes a more anxiety-provoking environment, resulting in sudden loss of control and the subsequent control of the emotions which can explain this relationship. We can deduce that the locus of control positively and significantly predicts pre-competitive anxiety (somatic anxiety and cognitive anxiety) 64% which leads us to conclude that the locus of control is considered an explanatory element of anxiety in pre-competitive situations, which confirms our first hypothesis. Our results agree with Arnaud et al. (2012) who found that the nature of the locus of control predicted 21.7% of the level of competitive anxiety in high-level tennis players. This could be explained by a difference in the scales used to assess control. Indeed, Arnaud et al. (2012) used the Levenson scale (Levenson and Ruef, 1992), while we used the Rotter scale (Rotter, 1966). In the study by Bloomfield et al. (2007) using young competitive swimmers, they measured the impact of the causal attributions of these children on their sports performance and observed in those who won their race a preferential internal allocation; at the same time, she also found in the same children, lower levels of cognitive anxiety (not found with somatic anxiety). Therefore, relationships between locus and cognitive anxiety and the relationship between locus and somatic anxiety differ. This is also true according to the extensive experiments conducted by Taylor (1987) who found that the two cognitive and somatic components of anxiety were only moderately correlated. In addition, according to Morris and Libiert in 1973 (Morris et al., 1981), cognitive anxiety reacts more to self-representations, social evaluation and social issues. On the contrary, the fear of electric shocks, for example, would selectively increase somatic anxiety.

While we have distinguished that the locus of control explains negatively and significantly 61% of self-confidence (when one variable decreases, the other increases and vice versa.) and can be interpreted as when the athlete is confident the level of the locus decreases (internal) and when the athlete is less confident the locus level rises (external). Obviously, confident people are optimistic, and think they have an influence on situations around them. These individuals also regulate their emotions more effectively, so highly trained soccer players who have an internal locus of control can control their emotions. In addition, they can resist anxiety, and this promotes their self-confidence unlike highly trained soccer players influenced by external control. Our reflection is consistent with that of Bandura (Bandura and Walters, 1977; Bandura, 2013) who indicates that self-confidence can be considered as a feeling of competence that generates expectations of success for future situations as well as personal effectiveness and this not only reduces anticipatory fears but incites to expend more efforts aimed at achieving the expected objective. Our results also agree with Patrick (Patrick et al., 2012) who suggests that subjects with an internal locus are more persevering, more confident, more independent and more resistant to failure unlike subjects with an external locus.

Our second hypothesis is verified through the observation of the scores presented in the results where the greater the athlete’s anxiety (cognitive anxiety) the more they present a high score in the locus of control. Therefore, they manifest an external attribution unlike the highly trained soccer players who are less anxious (cognitive anxiety) they have low scores in the locus of control and have internal attribution. In other words, we can state that when the athlete perceives that they can successfully meet the demands of competition, they then anticipate positive consequences as well as perceiving a causal relationship between their own actions and the results that ensue. This series of internal events leads to internal control. On the other hand, when an athlete perceives that they cannot meet the demands of the competition successfully they then anticipate negative consequences. In addition, the athlete may not perceive the existence of such a causal relationship and attributes reinforcements to luck or chance, this judgment leads to external control and confirms the definition of Morris et al. (1981) who stated that cognitive anxiety is characterized by conscious subjective feelings of apprehension and tension, caused by negative expectations of success or negative self-evaluations so it cannot have an internal locus. The results presented here reflect previous studies and agree with the findings of Burton’s (Burton, 1989) in his distinction between mastery of goals and competitive goals. The results recorded by Burton (Burton, 1989) show an association between reduced competitive anxiety and attributions towards internal causalities. In addition, Paquet (Paquet et al., 2006) showed that only the internal dimension of the locus of control and the desire for control are factors opposed to anxiety. Also, a recent American study by Holden et al. (2019) points out that highly trained soccer players who have an external locus of control feel they have little control over their situation. Gernigon et al. (2000) suggests that when this determinant has an internal origin, individuals engage in an activity in a free manner. When the origin is external, the actions respond to salient pressures from the environment.

This study is not without limitations. Firstly, although this research can be generalized with caution to other sports that include individuals with similar ages, further research using larger samples using different team sports (basketball, handball rugby) and individual sports (wrestling, judo, fencing.) may be required to confirm the findings. Secondly, the highly trained soccer players in our research sample were questioned in relation to a single competition, whereas the level of anxiety differs from one situation to another and from one competition to another. Thirdly, the way athletes of different maturation status would respond to sports activities has not yet been established. Hence future research investigated the association between locus of control and precompetitive anxiety in youth athltes with diffrent maturity statues (pre, *circa* and post PHV) are warranted. Fourthly, we did not study the association between the locus of control and precompetitive anxiety in athletes competing in different sports. Future similar studies should also investigate gender effects and differences associated with locus of control. Finally, checking out the relation between the above mentionned aspects and coaching behavior in relation to individual sport performance for future research, is also required.

## 5. Conclusion

The results obtained suggest that the levels of pre-competitive anxiety were explained through the levels of locus of control (especially cognitive anxiety which is predicted more than somatic anxiety) in highly trained cadet soccer players. In addition, the participants who are the most anxious (cognitive anxiety) have an external locus and the reverse is true for the less anxious. Given the absolute impact of lifestyle behaviors on mental health and quality of life, a multidisciplinary approach should be envisaged to understand the mechanisms explaining the rates of psychological issues (e.g., anxiety), and to develop the best methods (i.e., locus of control) to permit individuals to adhere to coping behaviors during soccer activity or boosting autonomous behavior. This statement should be the focus of research priority to support elite athletes against negative health consequences and soccer performance optimization.

From a practical point of view, we can provide coaches with information that can help predict pre-competitive anxiety. Coaches can implement mental preparation techniques for highly trained soccer players who tend more towards the external locus by working on self-esteem, and emotional control. Preparing highly trained soccer players on how to deal with their anxiety could prevent them from becoming overwhelmed when they feel powerless to change their situation during competition. In addition, the locus of control can be used in the context of identification and selection of young talent with the best aptitudes to face objectives. Further studies on anxiety and performance are required to improve the performance of highly trained soccer players. It would be interesting to compare the anxiety of highly trained soccer players and other highly competitive team sports and compare variables such as self-esteem, motivation, and other characteristics related to competition. The findings would provide a broader view of sport psychology in individual highly trained soccer players and team sports.

## Data availability statement

The original contributions presented in the study are included in the article/supplementary material, further inquiries can be directed to the corresponding author.

## Ethics statement

The study was conducted according to the latest version of the Declaration of Helsinki, and the protocol was fully approved by the Local Ethics Committee of the Higher institute of Sport and Physical Education of Ksar-Said (ISSEP Ksar-Said 17JS01) before the commencement of assessments. Legal guardians and participants provided written informed consent.

## Author contributions

IA, OC, CG, AK, and RG participated to the conception and design of the study. IA and OC were responsible for the testing. AK, CG, OC, and RG were responsible for the data collection and statistical analysis. IA, OC, CG, AK, RG, and JB were responsible for writing and finalization of the manuscript. All authors contributed to manuscript and approved the submitted version.

## Conflict of interest

The authors declare that the research was conducted in the absence of any commercial or financial relationships that could be construed as a potential conflict of interest.

## Publisher’s note

All claims expressed in this article are solely those of the authors and do not necessarily represent those of their affiliated organizations, or those of the publisher, the editors and the reviewers. Any product that may be evaluated in this article, or claim that may be made by its manufacturer, is not guaranteed or endorsed by the publisher.
