# Corrigendum: The relationship between locus of control and pre-competitive anxiety in highly trained soccer players

**DOI:** 10.3389/fpsyg.2023.1272127

**Published:** 2023-08-21

**Authors:** Imen Ben Amar, Chiraz Gomni, Oussama Gaied Chortane, Aymen Khemiri, Rania Ghouaiel, Julien S. Baker

**Affiliations:** ^1^High Institute of Sport and Physical Education, Manouba University, Tunis, Tunisia; ^2^Research Unit Sports Performance, Health and Society, ISSEP Ksar Saïd, Manouba, Tunisia; ^3^Philab Laboratory of Cultures, Technologies, and Philosophical Approaches, Tunis, Tunisia; ^4^Centre for Health and Exercise Science Research, Hong Kong Baptist University, Kowloon, Hong Kong SAR, China

**Keywords:** precompetitive anxiety, locus of control, internal locus, external locus, elite athletes

In the published article, an author name was incorrectly written as “Oussema Gaied”. The correct spelling is “Oussama Gaied Chortane”.

The citation for Oussama Gaied Chortane was incorrectly written as “Gaied O”, the correct spelling is “Chortane OG”.

The **Author contributions** attribution for Oussama Gaied Chortane was incorrectly written as “OG”, the correct spelling is “OC”.

## Author contributions

IA, OC, CG, AK, and RG participated to the conception and design of the study. IA and OC were responsible for the testing. AK, CG, OC, and RG were responsible for the data collection and statistical analysis. IA, OC, CG, AK, RG, and JB were responsible for writing and finalization of the manuscript. All authors contributed to manuscript and approved the submitted version.

The authors apologize for this error and state that this does not change the scientific conclusions of the article in any way. The original article has been updated.

